# Adaptive Immunity in Pulmonary Sarcoidosis and Chronic Beryllium Disease

**DOI:** 10.3389/fimmu.2020.00474

**Published:** 2020-03-18

**Authors:** Sarah A. Greaves, Shaikh M. Atif, Andrew P. Fontenot

**Affiliations:** ^1^Department of Medicine, University of Colorado Anschutz Medical Campus, Aurora, CO, United States; ^2^Department of Immunology and Microbiology, University of Colorado Anschutz Medical Campus, Aurora, CO, United States

**Keywords:** human, T cells, MHC, lung, T cell receptors, sarcoidosis

## Abstract

Pulmonary sarcoidosis and chronic beryllium disease (CBD) are inflammatory granulomatous lung diseases defined by the presence of non-caseating granulomas in the lung. CBD results from beryllium exposure in the workplace, while the cause of sarcoidosis remains unknown. CBD and sarcoidosis are both immune-mediated diseases that involve Th1-polarized inflammation in the lung. Beryllium exposure induces trafficking of dendritic cells to the lung in a mechanism dependent on MyD88 and IL-1α. B cells are also recruited to the lung in a MyD88 dependent manner after beryllium exposure in order to protect the lung from beryllium-induced injury. Similar to most immune-mediated diseases, disease susceptibility in CBD and sarcoidosis is driven by the expression of certain MHCII molecules, primarily *HLA-DPB1* in CBD and several *HLA-DRB1* alleles in sarcoidosis. One of the defining features of both CBD and sarcoidosis is an infiltration of activated CD4+ T cells in the lung. CD4+ T cells in the bronchoalveolar lavage (BAL) of CBD and sarcoidosis patients are highly Th1 polarized, and there is a significant increase in inflammatory Th1 cytokines present in the BAL fluid. In sarcoidosis, there is also a significant population of Th17 cells in the lungs that is not present in CBD. Due to persistent antigen exposure and chronic inflammation in the lung, these activated CD4+ T cells often display either an exhausted or anergic phenotype. Evidence suggests that these T cells are responding to common antigens in the lung. In CBD there is an expansion of beryllium-responsive TRBV5.1+ TCRs expressed on pathogenic CD4+ T cells derived from the BAL of CBD patients that react with endogenous human peptides derived from the plexin A protein. In an acute form of sarcoidosis, there are expansions of specific TRAV12-1/TRBV2 T cell receptors expressed on BAL CD4+ T cells, indicating that these T cells are trafficking to and expanding in the lung in response to common antigens. The specificity of these pathogenic CD4+T cells in sarcoidosis are currently unknown.

## Introduction

Granulomatous lung diseases represent a diverse set of disorders that are caused by both infectious and non-infectious agents that induce granuloma formation in the lung. Infectious organisms that induce granulomas include *Mycobacteria* sp. and various fungal organisms, while non-infectious etiologies include hypersensitivities, particulate exposures, and autoimmune reactions, among others [reviewed in (1)]. Because the clinical manifestations of many of these diseases are similar, the differential diagnosis is broad, and it is sometimes difficult to obtain diagnostic certainty. Sarcoidosis and chronic beryllium disease (CBD) are both non-infectious granulomatous lung diseases defined by the presence of non-caseating granulomas, comprised primarily of lymphocytes, epithelioid cells, giant cells, and macrophages (2–4). Although sarcoidosis can affect many organ systems, it involves the lungs in ~95% of cases (3), while CBD is typically limited to the pulmonary system.

Sarcoidosis is a disease of unknown etiology that occurs in individuals of all age, sex, and ethnic backgrounds. The highest prevalence of sarcoidosis is found in Sweden and the United States (5). CBD results from exposure to beryllium, which is a rare alkaline earth metal with exposures occurring through inhalation in the workplace, particularly in the United States (6). The adverse health effects of beryllium have long been described (7), and despite the implementation of beryllium exposure standards (8) cases of beryllium-induced disease continue to occur. The clinical manifestations of both CBD and sarcoidosis can be highly variable. In sarcoidosis, symptoms range from coughing to loss of lung function [reviewed in (9)]. Some patients develop an acute form of the disease that is self-resolving, while others develop a chronic condition that can substantially affect their quality of life. The average mortality rate for sarcoidosis patients is 3.6 per million, although this is highly variable depending on age, sex, and ethnicity (5). The first stage of beryllium-induced disease is beryllium sensitization (BeS), which involves beryllium-specific immune responses in the blood with no clinical manifestations (10). A subset of BeS patients eventually develop CBD, depending on quantity of beryllium exposure and genetic susceptibility of the individual (11, 12). If left untreated, CBD can progress to lung fibrosis, with one-third of patients historically progressing to respiratory failure (13).

Both diseases are characterized by an infiltration of activated CD4+ T cells in the lung. In CBD patients, these T cells recognize beryllium, while in sarcoidosis their specificity is unknown. Certain MHCII molecules, primarily *HLA-DPB1* in CBD and several *HLA-DRB1* alleles in sarcoidosis, increase disease susceptibility (14–17). Recently, there has also been evidence to suggest that B cells and antibody responses are involved in the pathogenesis of CBD and sarcoidosis (18–20). This review focuses on how different aspects of the immune system influence the progression of these diseases.

## Innate Immune Responses in CBD and Sarcoidosis

Innate immune cell populations are involved in propagating inflammation in both CBD and sarcoidosis (Table 1). Alveolar macrophages (AMs) comprise a major cell population in a healthy lung and are crucial for maintaining lung immunity (21). AMs are altered in sarcoidosis, with protein profiling revealing 80 differentially-expressed proteins in AMs of sarcoidosis patients compared to control subjects, including increased expression of two major phagocytic pathways (22). Activation of the mTORC1 pathway by deletion of the TSC2 gene in macrophages promoted excessive granuloma formation in mice (23). In sarcoidosis patients, mTORC1 activation and macrophage proliferation are associated with disease progression (23). Additionally, the number of monocytes undergoing phagocytosis in the blood of sarcoidosis patients is heightened compared to control subjects (24), and circulating monocytes also display higher TLR2 and TLR4 expression (25). Bronchoalveolar lavage (BAL) cells from sarcoidosis patients produce more TNF-α and IL-6 in response to TLR2 agonists than healthy controls, while PBMCs from sarcoidosis patients had impaired TLR2 responses (26, 27). Serum amyloid A (SAA) proteins are a group of acute phase inflammatory response proteins that are significantly elevated in the granulomas of sarcoidosis patients and localize to macrophages and giant cells within the granulomas (28). In a murine model of granulomatous lung inflammation SAA amplified inflammatory responses via TLR2 signaling (28). It has also recently been shown that SAAs promote Th17 induced inflammation (29), which will be discussed in the T cell section below. Certain MyD88 polymorphisms are also associated with the development of sarcoidosis (30). While these studies have led some to speculate that the abnormal inflammation in sarcoidosis is caused by aberrant monocyte and macrophage activity and TLR responses, the evidence for specific antigenic stimuli, as discussed below, indicates that there is an interplay between the innate and adaptive immune responses that drives the inflammation and granuloma formation.

**Table 1:** Immune characteristics of chronic beryllium disease and sarcoidosis.

	**Sarcoidosis**	**Chronic beryllium disease**
Innate Immunity	Increased TLR2 and TLR4 expression on monocytes Alveolar macrophages display increased phagocytosis Increased mTORC1 activation and macrophage proliferation PMBCs show impaired TLR2 responses MyD88 polymorphisms are associated with disease	Beryllium has adjuvant properties that prime innate immunity PMBCs and DCs have increased production of inflammatory cytokines Increased CD80 and CD86 on DCs Beryllium generates DAMPs to induce migration of DCs to lung
B cells/antibody responses	Increased IgA Anti-vimentin antibodies in BAL fluid	B cells recruited to lung dependent on MyD88 and organized into ELAsB cell depletion enhances lung injury
HLA susceptibility	HLA-DRB1*11:01 (US) HLA-DRB1*15:01 (US) HLA-DRB1*03:01 (Sweden)	HLA-DPB1*02:01 HLA-DPB1*17:01
Cytokine profiles	Increased Th1 polarized cytokines including TNF-α, IL-6, IFN-γ, IL-1, IL-2, IL-12	Increased Th1 polarized cytokines including IFN-γ, TNF-α, IL-2, IL-1
CD4+ T cells	CD4+ T cell alveolitis Majority Th1 polarized Th17 and Th17.1 CD4+ T cells also present Elevated PD-1 expression, dysfunctional TCR responses	CD4+ T cell alveolitis Majority Th1 polarized No evidence of Th2 or Th17 CD4+ T cells present Increased PD-1 and CTLA-4 expression Predominantly effector memory phenotype
Public TCRs	TCRs with TRAV12-1/TRBV2 are most frequent in HLA-DR3 Löfgren's syndrome patients	TRBV5.1 beryllium specific TCRs are expressed in majority of HLA-DP2 patients Public TCRs recognize HLA-DP2/beryllium/plexin A peptide complex

Similar to sarcoidosis, beryllium exposure and the development of granulomatous inflammation in CBD patients results from activation of both innate and adaptive immunity. Although beryllium is a part of an antigenic complex that generates an adaptive immune response in genetically-susceptible, beryllium-exposed workers, it also serves as an adjuvant to prime innate immunity (31–33). As compared to mice immunized with parasitic antigens alone, mice immunized with a combination of beryllium and parasitic antigens had enhanced control of the infection through increased production of IFN-γ in lymph nodes and spleen (34). PMBCs and dendritic cells (DCs) exhibit increased production of inflammatory chemokines after exposure to beryllium, and TNF-α was produced by beryllium-stimulated PBMCs independent of disease-associated HLA molecules (35, 36). In murine models of CBD, beryllium exposure promoted trafficking of DCs to lung-draining lymph nodes and enhanced expression of costimulatory molecules, CD80 and CD86, on DCs that was dependent on MyD88 signaling (37). Furthermore, it was recently demonstrated that exposure to beryllium hydroxide (Be(OH)_2_) resulted in the release of IL-1α and DNA into the airways, which subsequently acted as damage-associated molecular patterns (DAMPS) by engaging IL-1R1 and TLR9 to induce migration of DCs into lung-draining lymph nodes and the subsequent generation of memory CD4+ T cells (38). Thus, beryllium exposure has a crucial effect on innate cell function that contributes to disease development and progression.

## B Cell Involvement in CBD and Sarcoidosis

CBD and sarcoidosis have historically been thought of as T cell-driven inflammatory diseases. Until recently, the role of B cells in CBD was completely unknown. Our group recently demonstrated the importance of B cells in protecting the lung from beryllium oxide (BeO)-induced injury (Table 1). Using a murine model of CBD that mimics the human disease, we established that activated follicular B cells are recruited to the lung after beryllium exposure and are organized into ectopic lymphoid aggregates (ELAs) (20). B cell depletion eradicated these ELAs and enhanced lung injury, indicating that B cells play an important protective role in BeO-induced lung injury (20). In addition, BeO-induced B cell recruitment to the lung is dependent on MyD88 signaling (20). B cells also accumulate in the lungs of CBD patients, indicating that this protective role may translate to human disease (20). Further studies are needed to determine the exact mechanism of B cell-mediated protection in the lung after BeO exposure.

Although there is no evidence of B cells playing a direct role in disease pathogenesis in sarcoidosis, several studies have reported altered antibody responses in these patients. Particularly in patients with pulmonary sarcoidosis, there is a direct correlation between the number of T cells in the BAL and the proportion of BAL cells that secrete IgG, which is not apparent in control subjects (39). Additionally, increased numbers of memory B cells producing IgA are found in patients with sarcoidosis, raising the possibility that IgA could be involved in granuloma formation (40). Antibodies toward autoantigens have been found in only a small proportion of sarcoidosis patients, making it unclear whether autoimmune responses play a role in disease pathogenesis (18). Recent data show that in patients with an acute, self-resolving form of sarcoidosis, Löfgren's syndrome, there are increased quantities of anti-vimentin antibodies in the BAL fluid (19). Vimentin is a self-antigen that has been found in the lungs of sarcoidosis patients (41), although more validation is required to determine whether immune responses to vimentin are involved in disease initiation and progression. Overall, the role of B cells in sarcoidosis needs to be more thoroughly investigated to establish their role in this disease.

## T Cells in CBD and Sarcoidosis

**Figure 1:**
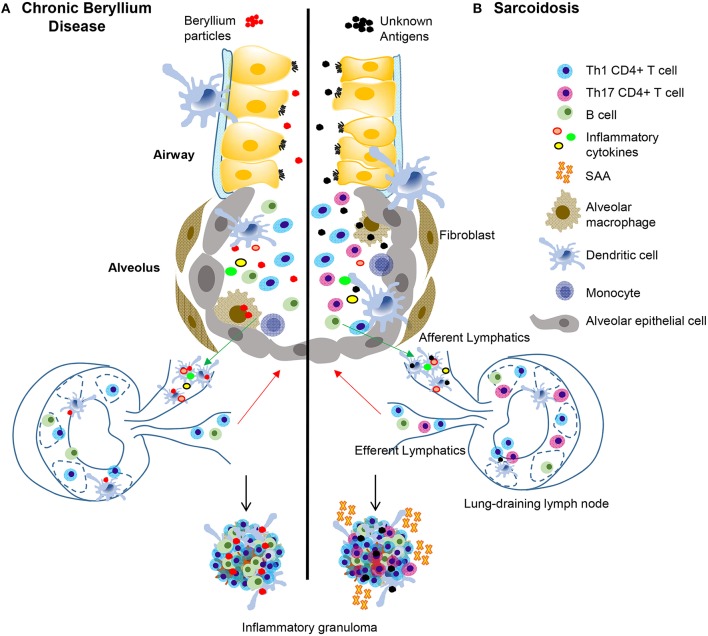
Immunopathogenesis of chronic beryllium disease and sarcoidosis. In CBD **(A)**, beryllium particles enter the lung and generate an immune response. In sarcoidosis **(B)** unknown antigens induce an immune response after entering the lung. In CBD, beryllium particles cause the release of DAMPs in the airways, which promote activation and migration of DCs. Either beryllium **(A)** or unknown antigens **(B)** are taken up by alveolar macrophages and dendritic cells in the lung and are transported to lung-draining lymph nodes. In the lymph nodes, antigen specific CD4+ T cells are stimulated by activated antigen-presenting cells expressing certain HLA molecules dependent on disease (see Table 1). T-B cell interactions also occur in the lymph nodes and commitment of B cells to antibody production takes place in the germinal centers. Antibodies and activated B and T cells circulate back to the lung to generate an adaptive immune response against the target antigen. In CBD, Th1 CD4+ T cells that display an effector memory phenotype are the primary cell type, but there is also an infiltration of activated B cells in the lungs that help form granulomatous structures **(A)**. In sarcoidosis and CBD, Th1 CD4+ T cells make up the majority of the cell population in the lungs. In sarcoidosis, but not CBD, there are also Th17 and Th17.1 cells present. SAAs are also present in pulmonary granulomas of sarcoidosis patients, but not CBD, where they contribute to the inflammatory milieu and induce proliferation of pathogenic, inflammatory Th17 cells. In both diseases, there is an abundance of Th1 inflammatory cytokines secreted by T cells and innate cells that propagate the inflammatory environment, including TNF-α, IFN-γ, IL-1, and IL-2.

A defining feature of both CBD and pulmonary sarcoidosis is an infiltration of pathogenic CD4+ T cells in the lung (42–47) (Table 1, Figure 1). CD4+ T cells in the BAL fluid of CBD patients are predominantly antigen-experienced T cells expressing CD45RO and lacking CD62L and CCR7 (48). Large numbers of these cells (in some cases, >30%) are responsive to beryllium sulfate (BeSO_4_) in culture, secreting Th1 cytokines, including IFN-γ and TNF-α and lesser quantities of IL-2 (48). CD4+ T cell responses to beryllium are dependent upon major histocompatibility complex class II (MHCII) molecules, but independent of CD28 costimulation, indicative of an effector memory T cell phenotype (48, 49). There is no evidence that beryllium-responsive Th2 or Th17 CD4+ T cells or CD8+ T cells are present in the lungs of CBD patients or that these cells play a role in beryllium-induced disease (48, 50).

CD4+ T cells from the BAL of sarcoidosis patients are also highly Th1 polarized. This enhanced Th1 polarization is driven by increased expression of inflammatory cytokines, such as IFN-γ, IL-1, IL-2, IL-6, IL-12, and TNF-α in addition to others, while Th2 cytokines have not been detected (51–53). Unlike CBD, there is no known inciting particle(s) or antigen(s) in sarcoidosis. However, in light of the infiltration of Th1-polarized CD4+ T cells in the lung, it is widely accepted that these T cells are trafficking to and proliferating in the lung in response to an unknown antigen. In contrast to findings in the BAL of sarcoidosis patients where a CD4+ T cell alveolitis (20–60% total cell count) exists (42), T cell lymphopenia in the peripheral blood is characteristic of the disease (54). CD4+ T cells with a Th17 phenotype and T cells expressing both IL-17 and IFN-γ (i.e., Th17.1 cells) have been found in lung tissue and BAL from patients with sarcoidosis (55, 56). A study recently demonstrated that there is a significant increase in CCR6+ Th17.1 cells in both the BAL fluid and mediastinal lymph nodes of sarcoidosis patients compared to controls and that these cells contribute to IFN-γ production (57). Additionally, Th17.1 cells in the BAL fluid are found in higher proportions in sarcoidosis patients with active disease vs. patients undergoing disease resolution, suggesting that these cells could be playing a role in disease progression (57). Interestingly, a recent study highlighted the importance of SAA in the differentiation of pathogenic Th17 cells in certain inflammatory conditions (29). SAA expression is increased in the lungs of sarcoidosis patients (28), so it is possible that SAAs contribute to the expansion of Th17 cells observed in these patients. Furthermore, SAAs in the lungs of sarcoidosis patients are significantly higher than that of CBD patients (28), which could explain the lack of Th17 cells in CBD.

As a consequence of constant antigen exposure in both sarcoidosis and CBD, pathogenic CD4+ T cells develop an anergic and/or exhausted phenotype as a mechanism to decrease chronic T cell activation and subsequent inflammation. Pulmonary CD4+ T cells from sarcoidosis spontaneously secrete IL-2 *ex vivo*, but when given TCR stimulation, these cells express less IL-2 and IFN-γ relative to CD4+ T cells from various other lung diseases and healthy control subjects, consistent with an anergic/exhausted phenotype (58). Additionally, sarcoidosis CD4+ T cells have a reduced proliferative capacity and higher levels of apoptosis, along with increased PD-1 expression, which is often associated with persistent antigen exposure to limit chronic T cell activation (59, 60). A recent study demonstrated that PD-1 expression was highest on sarcoidosis CD4+ T cells with a Th17 phenotype (61). Furthermore, PD-1+ Th17 CD4+ cells produce high levels of TGFB-1, a key factor in the development in fibrosis (61). PD-1 blockade significantly reduced TGFB-1 produced by Th17 CD4+ T cells from sarcoidosis patients and reduced their ability to induce collagen-1 production in a fibroblast cell line (61). These data demonstrate that T cell dysfunction in sarcoidosis is seemingly due to persistent antigen exposure, thus discovering the etiologic antigens driving this disease will be important for therapies directed at reducing antigen exposure.

BAL CD4+ T cells in CBD also have elevated PD-1 expression and blockade of the PD-1 pathway increases proliferation of beryllium-responsive CD4+ T cells (62). Interestingly, a subsequent study showed that CTLA-4, another T cell co-inhibitory receptor, was upregulated on BAL CD4+ T cells in CBD, but its expression was not capable of reducing beryllium-stimulated T cell proliferation (63). Taken together, these findings show that persistent antigen exposure drives an exhausted T cell phenotype in both CBD and sarcoidosis. Despite the immune systems attempt to dampen T cell-mediated immune activation, the cells are still able to promote chronic inflammation and disease progression in non-infectious granulomatous lung disease.

## Genetic Susceptibility to CBD and Sarcoidosis

Similar to most immune-mediated diseases, MHCII molecules are strongly associated with both sarcoidosis and CBD (Table 1). Saltini et al. (44) demonstrated that BAL CD4+ T cells from CBD patients recognize beryllium in an MHCII-restricted manner. Genetic susceptibility was strongly linked to HLA-DPB1 alleles with a glutamic acid (E) at position 69 of the β-chain (βGlu69), with the most prevalent βGlu69-containing allele being HLA-DPB1^*^02:01 (14). Multiple studies have corroborated these findings, documenting the presence of βGlu69-containing *DPB1* alleles in 73–95% of BeS and CBD patients compared to 30–48% of exposed controls [reviewed in (64)]. A differential risk of disease development is also associated with certain rare βGlu69-containing *DPB1* alleles, such as HLA-DPB1^*^17:01 (15, 65–67). Thus, CBD is a classic example of a disorder resulting from gene-by-environment interactions, where both components are required for the development of granulomatous inflammation. In this regard, the probability of CBD increases with HLA-DP βGlu69 copy number and increasing workplace exposure to beryllium (12), suggesting that genetic and exposure factors may have an additive effect on the risk of disease development (11).

Similar to CBD, genetics and environmental exposures are involved in the initiation of sarcoidosis. Because sarcoidosis is a more heterogeneous disease that occurs worldwide and the etiologic antigen(s) are currently unknown, genetic studies are important to determine common disease characteristics. However, many genetic studies involving sarcoidosis patients demonstrate that even commonalities among subsets of patients differ between ethnic and regional groups, suggesting that there are multiple factors involved in the progression of sarcoidosis. A large-scale ACCESS study that collected patient data in the United States from 1996 to 1999 demonstrated that siblings of sarcoidosis patients are about 5-fold more likely to develop the disease than unrelated control subjects (68), suggesting that genetic factors influence disease susceptibility. As suspected by the accumulation of CD4+ T cells in the BAL of sarcoidosis subjects, the dominant genetic associations in sarcoidosis are linked to HLA. While both HLA class I and class II genes are associated with the development of sarcoidosis, the strongest correlations are shown with class II genes, although these differ between ethnic and regional groups [reviewed in (69)]. In the United States, HLA-DRB1^*^11:01 and HLA-DRB1^*^15:01 are both associated with sarcoidosis (16). The most striking genetic association in sarcoidosis is present in Löfgren's syndrome in Scandinavian patients. Löfgren's syndrome is an acute form of sarcoidosis associated with a favorable prognosis that presents with a specific set of inflammatory symptoms including bilateral hilar lymphadenopathy, fever, erythema nodosum, and ankle arthritis (70). The majority of Löfgren's syndrome patients express HLA-DRB1^*^03:01, with most of these patients resolving their disease within 2 years (70). Specific αβTCR variable region genes are also highly associated with Löfgren's syndrome, as discussed below. These studies clearly demonstrate that genetic susceptibility due to expression of certain MHCII molecules in both CBD and sarcoidosis is a crucial aspect of disease pathogenesis.

## Public T Cells in BAL of CBD and Sarcoidosis Driving Disease Pathogenesis

As discussed in the previous section, specific MHCII expression is crucial for disease development in both CBD and sarcoidosis. CD4+ T cells recognize peptide epitopes in an MHCII-restricted manner (71). In CBD, MHCII molecules require beryllium for antigen presentation to pathogenic T cells (48). The positively-charged beryllium particle is coordinated in the MHCII groove along with specific naturally-occurring peptides to complete the beryllium-dependent T cell ligand (72, 73). BAL CD4+ T cells from CBD patients have unique oligoclonal populations that are enriched for certain TCR β-chain motifs (46, 74). Furthermore, there are public (i.e., expressed in the majority of HLA-DP2-expressing CBD patients) beryllium-responsive TCRβ variable region (TRBV) 5.1+ TCRs expressed on CD4+ T cells derived from the BAL of CBD patients, and the frequency of these public beryllium-responsive TCRs inversely correlate with loss of lung function, suggesting that these public T cells are pathogenic in nature (75). Falta et al. (76) discovered beryllium-dependent mimotopes (i.e., peptides that mimic the naturally-occurring epitope) that bound to HLA-DP2 in the presence of beryllium and are recognized by pathogenic TRBV5.1+ CD4+ T cells expanded in the lungs of HLA-DP2+ CBD patients. Our group further discovered that an endogenous human peptide derived from plexin A proteins binds to the HLA-DP2/Be complex and stimulates these same pathogenic CD4+ T cells from CBD patients (76). CD4+ T cells specific for the HLA-DP2-plexin A4/beryllium complex comprise ~5% of beryllium-responsive CD4+ T cells in the lung (76).

Although the inciting antigen(s) in sarcoidosis is unknown, there is strong evidence suggesting that the disease is driven by antigen-specific CD4+ T cells in the lungs of patients. The most conclusive evidence comes from Löfgren's syndrome. The majority of Löfgren's syndrome patients in Sweden express HLA-DRB1^*^03:01 (HLA-DR3) (17), and expression of HLA-DR3 was strongly associated with disease resolution and an excellent prognosis (70). Additionally, HLA-DR3-expressing Löfgren's syndrome patients exhibit oligoclonal expansions of CD4+ T cells in the BAL that express TCRα variable region (TRAV) 12-1, and the quantity of these cells in the BAL correlates with disease remission (77–79). Importantly, this expansion of TRAV12-1+ CD4+ T cells is only seen in patients with active disease, and upon disease resolution, these cells are greatly reduced, highlighting their importance in disease pathogenesis (80). Using deep sequencing approaches, our group recently demonstrated that TRAV12-1 preferentially pairs with TCRβ variable region (TRBV) 2 in BAL CD4+ T cells from Löfgren's syndrome patients and that TRAV12-1 and TRBV2 are the most expanded variable regions relative to control subjects (81). Additionally, there are specific TRAV12-1/TRBV2 CDR3 motifs expressed on BAL CD4+ T cells of multiple Löfgren's syndrome patients, indicating that these T cells are trafficking to and expanding in the lung in response to the same antigen.

## Antigenic Drivers of Sarcoidosis

One of the most active areas of sarcoidosis research is focused on determining the etiologic antigens that drive the recruitment of CD4+ T cells to the lungs of sarcoidosis patients. One problem hindering this endeavor is the lack of understanding of the origin of the inciting antigens, whether it is a foreign or self-antigen, or even a product of environmental exposure. Although there is some evidence for each of these categories, nothing has been conclusive enough to determine a cause of disease. The most detailed studies involving an autoantigen focus on vimentin, a filamentous protein of which the cytoskeleton is composed (82). Vimentin has been eluted from HLA-DR molecules expressed on BAL cells from some patients with sarcoidosis, and vimentin-specific autoantibodies are found in BAL fluid of sarcoidosis patients (19, 41, 83). More validation is required to determine whether immune responses to vimentin are involved in disease initiation and progression.

Due to the similarities between the clinical features of sarcoidosis and certain pulmonary infections, it has long been thought that there may be an infectious agent driving sarcoidosis etiology. The most substantial evidence linking a microorganism to sarcoidosis pathogenesis involves *Mycobacteria sp*. Several meta-analyses demonstrate that the odds of finding mycobacterial DNA by PCR in the lungs of sarcoidosis patients is ~5–20% greater than in control subjects (84, 85). However, the data collected in these studies is very heterogeneous, and *Mycobacteria sp*. are not detected in the majority of sarcoidosis patients.

To investigate whether there were any functional implications that mycobacterial infections induce sarcoidosis, several groups have measured T cell responsiveness to mycobacterial candidate antigens in sarcoidosis patients. A higher number of T cells in the blood of sarcoidosis patients produce IFN-γ after stimulation with a *M. tuberculosis* antigen catalase peroxidase (mKatG) (86). Additionally, T cells derived from the BAL fluid of sarcoidosis patients respond to mKatG along with an additional mycobacterial antigen, early secreted antigen protein (ESAT-6) (87). Furthermore, one study detected protein derived from mKatG in ~50% of tissue samples from sarcoidosis patients (88), and IgG antibodies were detected in ~50% of sarcoidosis serum samples (88). Despite this evidence, sarcoidosis patients show no signs active mycobacterial infections even during immunosuppressive treatments. There are also no mycobacterial-specific CD4+ T cell clones identified from sarcoidosis patients to date.

*Cutibacterium acnes* (previously *Propionibacterium acnes*) infections are also associated with sarcoidosis. *C. acnes* is a commensal bacteria that is commonly found on the skin of healthy individuals. By quantitative PCR, DNA from *C. acnes* is found in the majority of lymph nodes obtained from sarcoidosis patients (89). However, *C. acnes* is also found in a large percentage of lymph nodes isolated from healthy control subjects (90), making it difficult to ascertain whether the presence of *C. acnes* in sarcoidosis patients is a consequence of the disease or normal colonization of commensal bacteria.

In addition to infectious agents, it has also been postulated that there are environmental factors involved in the progression of sarcoidosis. Stage I sarcoidosis occurs more frequently in the Spring, and the lowest incidence of disease diagnosis occurs in the Winter, suggesting that airborne allergens or other seasonal particulates may be involved in disease progression (91). There are positive associations between the development of sarcoidosis and certain environmental and occupational exposures, such as insecticides and mold/mildew (92). “Sarcoid-like” granulomatous pulmonary disease occurred in rescue workers that were exposed to airborne particulates during the World Trade Center collapse (93). Recently, it was reported that patients exposed to occupational silica during work in the iron production industry in Sweden have a significantly higher incidence of sarcoidosis than unexposed individuals (94). Whether these exposures are driving sarcoidosis by activating specific CD4+ T cells in the lung or are merely inducing an inflammatory environment that makes individuals more susceptible to disease is currently unknown.

## Conclusions

The immunology of sarcoidosis and CBD are quite similar, based on a predominance of CD4+ T cells in the lung, a Th1 polarized immune response, and a pathologic hallmark of granulomatous inflammation. As such, the presence of a known antigen in CBD can further our understanding of the driving factors involved in sarcoidosis pathogenesis. In CBD, our group has performed unbiased antigen discovery approaches that focused on the related and expanded CD4+ T cell subsets in the BAL of patients with active disease and have delineated several beryllium-dependent T cell ligands. It is possible that a similar unbiased antigen discovery approach utilizing expanded CD4+ T cell clones in the lungs of sarcoidosis patients may lead to the discovery of the inciting antigens that drive CD4+ T cell alveolitis and granulomatous inflammation in sarcoidosis.

## Author Contributions

SG, SA, and AF wrote the article. SA contributed to designing the figure and table for the article. All authors contributed to article revisions.

### Conflict of Interest

The authors declare that the research was conducted in the absence of any commercial or financial relationships that could be construed as a potential conflict of interest.

## Abbreviations

CBD: chronic beryllium disease
AM: alveolar macrophage
DC: dendritic cell
DAMP: damage associated molecular pattern
BeO: beryllium oxide
BAL: bronchoalveolar lavage
ELA: ectopic lymphoid aggregate
TRAV: TCR alpha variable region
TRBV: TCR beta variable region
SAA: serum amyloid A.
